# Effects of Acute Citrulline Malate Supplementation on Repeated 100 m Sprint Performance in Trained Sprinters: A Randomized Crossover Study

**DOI:** 10.3390/sports14040143

**Published:** 2026-04-07

**Authors:** Ryo Yamanaka, Kenichi Abe, Ryo Kojima, Tsubasa Nagai, Yoichi Maekawa

**Affiliations:** 1 Faculty of Human Health, Kurume University, Fukuoka 839-8502, Japan; 2 Faculty of Agro-Food, Niigata Agro-Food University, Niigata 959-2702, Japan; kenichi-abe@nafu.ac.jp (K.A.); yoichi-maekawa@nafu.ac.jp (Y.M.)

**Keywords:** repeated sprint performance, track and field, sports nutrition, perceived exertion, blood lactate

## Abstract

Strategies that support repeated high-intensity sprint performance are of considerable interest in competitive sprinting and team sports; however, evidence regarding acute citrulline malate (CM) supplementation during recovery intervals remains limited. This study examined the effects of acute CM supplementation on repeated 100 m sprint performance and rating of perceived exertion (RPE) in trained sprinters. Eleven trained male collegiate sprinters (100 m personal best: 11.22 ± 0.47 s; range: 10.35–12.16 s) completed randomized, double-blind, crossover trials (CM vs. placebo) on separate days. Each trial consisted of two maximal 100 m sprints separated by an 80–90 min recovery interval, during which 8 g of CM or placebo was ingested. Sprint performance was primarily evaluated using wind-adjusted 100 m sprint time based on Mureika’s model. A significant condition × trial interaction was observed for wind-adjusted sprint time (*p* = 0.010), with a greater improvement in the CM condition (*p* = 0.008). RPE (Borg 6–20 scale) before the second sprint was lower (*p* = 0.004) following CM supplementation. These findings suggest that acute CM supplementation may modestly support repeated sprint performance under extended recovery conditions; however, the results should be interpreted with caution.

## 1. Introduction

Short-distance sprint performance is influenced by both energetic and intramuscular factors. During an all-out 100 m sprint lasting approximately 10 s, energy is supplied primarily by the ATP–phosphocreatine system, with additional contributions from anaerobic glycolysis and only a minor aerobic contribution [1]. High-intensity sprinting also induces substantial metabolic disturbances, including reductions in pH and the accumulation of ammonia, which may impair muscle function and limit performance [2]. In competitive sprint events, athletes are often required to complete multiple rounds (e.g., heats, semifinals, and finals) within the same day, highlighting the importance of reproducing maximal sprint performance after a limited recovery interval.

Citrulline malate (CM), a compound consisting of citrulline bound to malate, has attracted interest as a potential ergogenic aid. After ingestion, citrulline is converted to L-arginine via the argininosuccinate pathway, which may support nitric oxide (NO) production [3]. CM has also been proposed to influence ammonia clearance and metabolic regulation during exercise [4,5], although the extent to which these mechanisms contribute to sprint performance remains unclear. Importantly, a previous pharmacokinetic study reported that plasma concentrations of NO metabolites (NO_x_) peak approximately 60–90 min after ingestion [6], suggesting that supplementation during a competition-like recovery interval may be practically relevant.

Most previous studies on citrulline-based supplementation have focused on submaximal aerobic or resistance exercise. For example, citrulline supplementation has been reported to improve cycling time-trial performance and oxygen uptake kinetics [7,8], while acute CM ingestion has also been associated with improved resistance exercise performance and reduced ratings of perceived exertion (RPE) [9,10]. More broadly, nutritional interventions targeting repeated high-intensity performance have also shown mixed findings depending on the exercise model and recovery structure. For example, creatine-based supplementation has improved repeated sprint performance in some treadmill- and field-based protocols [11,12], whereas interventions targeting related nitric oxide pathways do not necessarily enhance anaerobic performance in trained athletes [13]. However, evidence regarding the effects of CM on true maximal sprint performance remains limited. Although Faria and Egan reported improvements in repeated sprint running after short-term CM ingestion [14], their protocol likely involved a substantial aerobic contribution [15], making direct application to maximal 100 m sprinting difficult.

To date, no study has examined whether acute CM ingestion during a prolonged recovery interval influences performance in a second maximal 100 m sprint in trained sprinters. This question has direct practical relevance to track-and-field competition, in which athletes may be required to reproduce maximal sprint performance after 60–120 min of recovery between rounds. Therefore, the present study aimed to investigate whether acute CM ingestion during the recovery interval between two maximal 100 m sprint trials influences second-trial sprint performance, blood lactate responses, and RPE in trained sprinters. It was hypothesized that CM supplementation would result in a greater improvement in second-trial sprint performance than placebo, accompanied by higher post-exercise blood lactate concentrations and lower perceived exertion before the second sprint.

## 2. Materials and Methods

### 2.1. Participants

This study included eleven male collegiate sprinters specializing in the 100 m event. Their mean (±standard deviation [SD]) age, height, and body mass were 20.5 ± 1.2 years, 171.7 ± 3.8 cm, and 67.0 ± 4.3 kg, respectively. Their personal best performance in the 100 m sprint and World Athletics points were 11.22 ± 0.47 s (range: 10.35–12.16 s) and 870.5 ± 117.7 (range: 576–1089), respectively. Based on the Participant Classification Framework [16], all participants were classified as Tier 3 (highly trained). The participants had 8.3 ± 1.5 years of sprint-specific training experience and trained six days per week. All participants were free from illness or musculoskeletal injuries at the time of the study.

Before participation, the purpose and procedures of the study were explained both verbally and in writing, and written informed consent was obtained. The study protocol was reviewed and approved by the Research Ethics Committee of Kurume University in accordance with the 1964 Declaration of Helsinki (Approval No. 518). Participants were instructed to refrain from caffeine, alcohol, and all ergogenic supplements (e.g., creatine, nitrate, β-alanine, and bicarbonate) for 72 h before each trial to avoid confounding effects, as recommended in sprint-related supplementation research.

An a priori power analysis was conducted using G*Power 3.1. Based on the effect sizes (Set 2′: d ≈ 0.8, Set 3′: d ≈ 1.0) calculated from the results of a previous study [17] for improvements in repeated performance during the latter sets of resistance exercise, a paired-samples *t*-test (two-tailed, α = 0.05, 1 − β = 0.80) indicated that 10 participants would be sufficient to detect a statistically significant effect. The study sample (*n* = 11) met this requirement. Although this effect size was derived from resistance exercise rather than sprinting, no prior studies have reported effect sizes for repeated 100 m sprint performance after citrulline supplementation. Therefore, the present study adopted the closest available evidence related to repeated high-intensity efforts. However, we acknowledge that this approach may not perfectly represent the variability of sprint performance.

### 2.2. Procedures

All testing sessions were performed on the same all-weather outdoor track. in the same lane, at the same time of day for each participant, and under comparable environmental conditions (temperature: 28.7–32.5 °C; relative humidity: 54.3–58.2%; no precipitation). Wind speed was also monitored during each trial, and wind-adjusted sprint times were calculated to further improve comparability across testing sessions. Each participant completed both the CM and placebo trials in a counterbalanced order, with the assignment determined using a computer-generated random sequence. To ensure blinding, both CM and placebo were prepared and administered in an identical manner using edible wafer paper, and the allocation was concealed from both participants and investigators throughout data collection.

**Figure 1:**
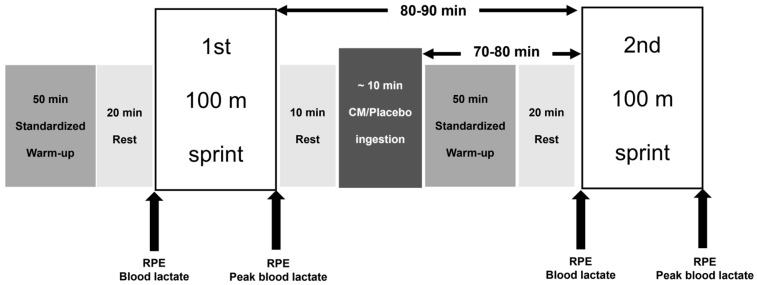
Schematic overview of the experimental protocol.

On each test day, the participants performed a standardized 50 min warm-up, consisting of 5 min of jogging, stretching, 10 min of sprint drills, and two 50–60 m sprints at approximately 80–90% of maximal effort. After a 20 min rest, they performed the first 100 m sprint trial at maximal effort from a standing start. All sprints were initiated from a standing start to ensure consistency and to avoid variability associated with block settings. Following a 10 min rest, they ingested either CM or placebo over approximately 10 min. They subsequently completed the same 50 min standardized warm-up protocol, rested for another 20 min, and then performed the second 100 m sprint trial from a standing start (Figure 1). The same standardized warm-up was repeated before the second sprint to mimic competition-like conditions in which sprinters typically perform a re-warm-up prior to subsequent rounds and to ensure a comparable state of readiness before each sprint. Although a shorter activation routine may also have been possible, the present design prioritized standardization and ecological validity. Accordingly, the second sprint trial was conducted approximately 70–80 min after supplementation.

Participants were explicitly instructed to perform each 100 m trial with maximal effort (all-out), and any form of pacing was strictly prohibited. High-speed video recordings were used to confirm that all participants ran with maximal intent and without pacing strategies. Participants were asked to refrain from strenuous exercise for 24 h before testing and to consume identical meals the night before and the morning of both trials. Dietary intake recorded on Day 1 was replicated on Day 2 under supervision. Participants also maintained identical sleep schedules before both testing sessions.

On each trial day, participants performed two maximal 100 m sprints under either the citrulline malate (CM) or placebo condition. A standardized 50 min warm-up and 20 min rest period preceded the first sprint. Participants then rested for 10 min before ingesting CM or placebo over approximately 10 min. A second standardized 50 min warm-up and 20 min rest period were completed prior to the second sprint. Accordingly, the interval from the start of supplementation to the second sprint was approximately 70–80 min, whereas the interval between the first and second sprints was approximately 80–90 min. RPE and blood lactate concentration were obtained before and after each sprint, with peak blood lactate concentration determined after each trial.

### 2.3. Supplementation Protocol

Participants ingested 8 g of commercially available CM (MYPROTEIN, Manchester, UK; citrulline-to-malate ratio of 2:1) or 8 g of microcrystalline cellulose (placebo), each encapsulated in edible wafer paper to mask appearance and taste, with 350 mL of water. Both participants and investigators were blinded to the assigned condition. The 8 g dosage was selected based on prior studies demonstrating ergogenic effects at this level [17]. Plasma NOx concentrations peak approximately 60 min after CM ingestion [6]; therefore, the second sprint was scheduled 70–80 min after ingestion to correspond with the expected peak in circulating NO availability.

### 2.4. Measurements

Sprint performance was measured using photoelectric timing gates (SPEED TECH S-001, Xuanying, Dongguan, China) positioned at the start and finish lines, with a precision of 0.001 s. Raw 100 m sprint time obtained from the timing gates was treated as a secondary performance outcome. Moreover, because hand movements can sometimes prematurely trigger the light beam, a high-speed video camera (Lumix DC-FZ300, Panasonic, Tokyo, Japan) was used to record each sprint in panning mode. The video data were analyzed using motion analysis software (Kinovea version 2023.1, open source; https://www.kinovea.org/). The number of frames from the torso crossing the start line to that crossing the finish lines was counted, and the total frame count was divided by the frame rate (239.76 Hz) to calculate sprint time. These video data were used to verify timing-gate measurements and identify any premature beam interruptions. A comparison between the sprint time obtained from the photocell system (11.246 ± 0.461 s) and those derived from the high-speed video recordings (11.247 ± 0.459 s) across trials (*n* = 44, subjects: 11, conditions: 2, 100 m sprint trials: 2) revealed no significant difference (*p* = 0.894; paired-sample t-test). Therefore, sprint times measured by the photocell system were used for the main analyses in this study. Because all sprint trials were conducted outdoors, average wind velocity was measured during each 100 m trial using an anemometer (BT-100, BTMETER, China). To standardize the results, wind-corrected sprint times were adjusted to 0 m·s^−1^ wind speed using the correction formula proposed by Mureika (Equation (9), [18]). Given the known influence of wind on sprint performance, the wind-corrected 100 m sprint time was defined a priori as the primary performance outcome of the study.

Capillary blood samples were obtained from the fingertip immediately before the first sprint trial (baseline), immediately before the second sprint trial, and every minute after each sprint until two consecutive decreases were observed. The highest value during that period was defined as the peak blood lactate concentration. Blood lactate concentration was determined using a portable analyzer (Lactate Pro 2, Arkray, Kyoto, Japan).

Subjective exertion was assessed immediately before and after each sprint using the Borg 6–20 scale [19]. RPE before each sprint was recorded to provide an index of the participants’ perceived readiness and residual exertional state prior to each sprint trial.

### 2.5. Statistical Analyses

All statistical analyses were performed using SPSS Statistics version 26.0 (IBM Corp., New York city, NY, USA). Data are presented as mean ± SD. The normality of all dependent variables was verified using the Shapiro–Wilk test. The primary performance outcome was wind-adjusted 100 m sprint time, whereas raw sprint time was treated as a secondary performance outcome. Two-way repeated-measures analysis of variance (ANOVA) (condition × trial) was used to examine the effects of supplementation on sprint time, blood lactate concentration, and RPE. When a significant interaction was detected, paired-sample t-tests were conducted for simple comparisons between conditions or trials. The significance level was set at *p* < 0.05. Effect sizes for ANOVA were reported as partial eta-squared (η^2^p), interpreted as small = 0.01–0.05, medium = 0.06–0.13, and large ≥ 0.14 [20]. Effect sizes for t-tests were calculated as Cohen’s d, interpreted as small = 0.20–0.49, medium = 0.50–0.79, and large ≥ 0.80 [20]. Accordingly, sprint performance was primarily interpreted based on wind-adjusted sprint time, with raw sprint time used as a secondary field-based reference measure.

## 3. Results

Shapiro–Wilk tests demonstrated that sprint time and blood lactate concentration met the assumption of normality (*p* > 0.05). RPE showed a slight deviation from normality (*p* = 0.019); however, repeated-measures ANOVA is considered robust to such deviations, and therefore parametric analyses were conducted.

### 3.1. 100 m Sprint Time

**Figure 2:**
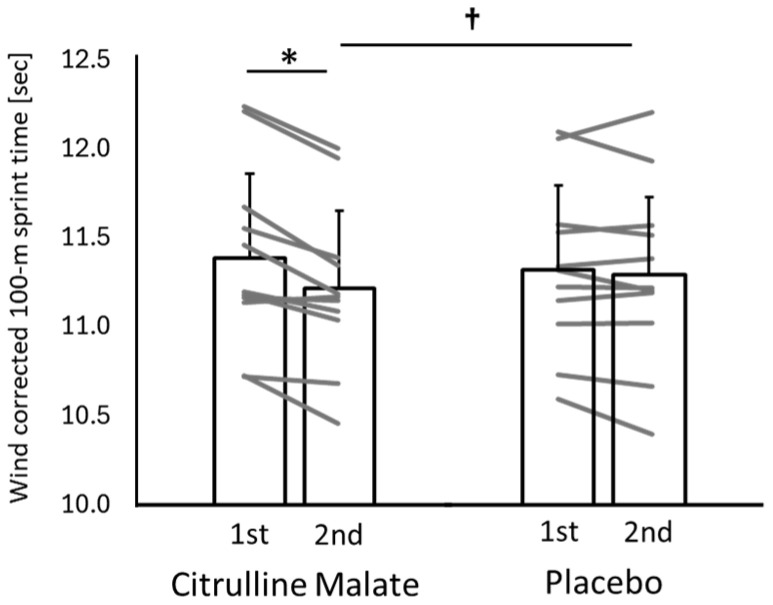
Wind-adjusted 100 m sprint times in the citrulline and placebo conditions across two trials. Individual participant data are also shown to illustrate inter-individual variability and response consistency across conditions. * indicates a significant difference between trials within the same condition. † indicates a significant difference between conditions at the second trial.

Figure 2 shows the 100 m sprint times corrected for wind velocity using Mureika’s model [18]. Wind-adjusted sprint time was treated as the primary performance outcome. A two-way repeated-measures ANOVA (condition × trial) revealed a significant interaction effect (F(1,10) = 10.108; *p* = 0.010; η^2^p = 0.517), with a large effect size. No significant difference was found between conditions in the first trial; however, in the second trial, sprint time was significantly shorter in the CM condition than in the placebo condition (*p* = 0.049; d = −0.16: very small effect). The improvement in sprint time from the first to the second trial was significantly greater in the CM condition (0.173 ± 0.125 s) than in the placebo condition (0.029 ± 0.096 s) (*p* = 0.008; d = 1.29: large effect). Within-condition analyses showed that the placebo condition exhibited no significant change, whereas the CM condition demonstrated a significant improvement in the second trial (*p* = 0.001; d = 0.38: small effect).

**Figure 3:**
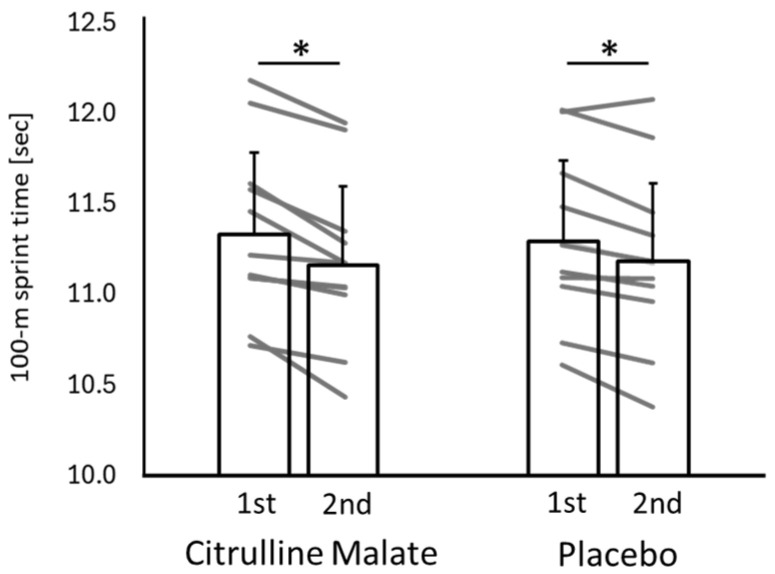
Raw 100 m sprint times in the citrulline and placebo conditions across two trials. Individual participant data are also shown to illustrate inter-individual variability and response consistency across conditions. * indicates a significant difference between trials within the same condition.

Figure 3 shows the raw 100 m sprint times for both conditions across the two trials. As a secondary performance outcome, a two-way repeated-measures ANOVA (condition × trial) revealed a significant interaction effect (F(1,10) = 5.478; *p* = 0.041; η^2^p = 0.354), indicating a large effect size. Although between-condition differences at each trial were not significant, the CM condition (0.173 ± 0.107 s) exhibited a greater improvement from the first to the second trial compared with the placebo condition (0.109 ± 0.085 s) (*p* = 0.041; d = 0.49: small effect). Under both conditions, sprint times significantly improved from the first trial to the second trial (CM: *p* < 0.001; d = 0.39; placebo: *p* = 0.002; d = 0.24), although the effect sizes were small. These findings should be interpreted cautiously and as secondary to the wind-adjusted sprint-time analysis.

### 3.2. Blood Lactate Concentration

**Figure 4:**
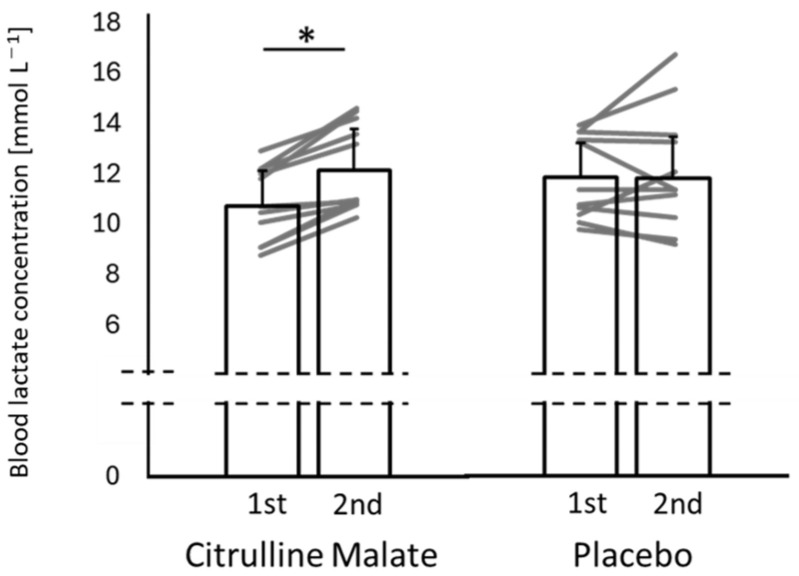
Peak blood lactate concentrations after the 100 m sprints in in the citrulline and placebo conditions. Individual participant data are also shown to illustrate inter-individual variability and response consistency across conditions. * indicates a significant difference between trials within the same condition.

No significant differences were observed between the two conditions in the resting values prior to either trial (CM: first trial = 1.40 ± 0.17, second trial = 1.49 ± 0.15 mmol·L^−1^; placebo: first trial = 1.35 ± 0.14, second trial = 1.47 ± 0.17 mmol·L^−1^) (first trial: *p* = 0.451; second trial: *p* = 0.840). Figure 4 shows the peak blood lactate concentrations after the 100 m sprint trials under both conditions. A significant interaction between condition and trial was observed (F(1,10) = 9.886; *p* = 0.010; η^2^p = 0.497: large effect). Although no significant differences were found between the conditions in either trial, the change in the peak lactate concentration from the first to the second trial was significantly greater in the CM condition (1.41 ± 0.73 mmol·L^−1^) than in the placebo condition (0.04 ± 1.36 mmol·L^−1^) (*p* = 0.010; d = 1.23: large effect). Within-condition analyses showed that the peak lactate concentration did not change significantly in the placebo condition, whereas it increased significantly in the CM condition during the second trial (*p* = 0.001; d = 0.92: large effect).

### 3.3. Rating of Perceived Exertion

**Figure 5:**
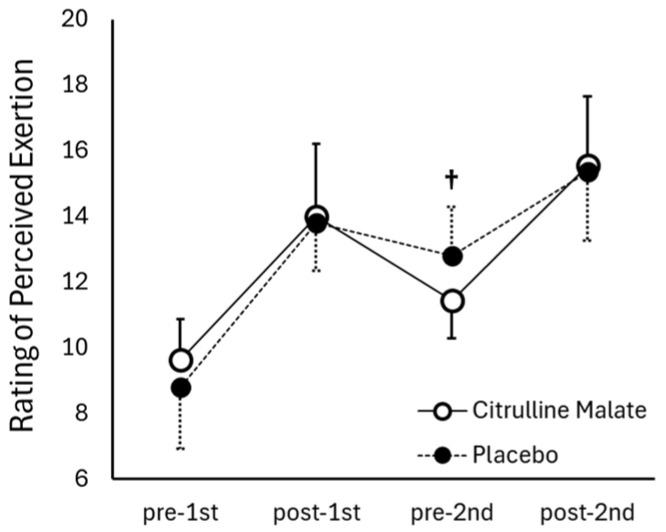
Perceived leg fatigue before and after each 100 m sprint in the citrulline and placebo conditions. † indicates a significant difference between conditions at the trial.

Figure 5 shows the changes in RPE across conditions and time points. A two-way repeated-measures ANOVA with condition (CM, placebo) and time (pre-first trial, post-first trial, pre-second trial, post-second trial) revealed a significant condition × time interaction (F(3,30) = 3.125; *p* = 0.040; η^2^p = 0.172: large effect). No significant between-condition differences were observed at pre-first trial, post-first trial, or post-second trial (*p* > 0.05). However, immediately before the second sprint, the CM condition (11.5 ± 1.2) showed significantly lower RPE scores than the placebo condition (12.8 ± 1.5; *p* = 0.004; d = 1.03: large effect), indicating reduced perceived exertion prior to the second sprint under CM supplementation.

## 4. Discussion

The present study investigated the effects of acute citrulline malate (CM) ingestion during the recovery interval between two maximal 100 m sprints in trained sprinters. The principal findings were that CM supplementation was associated with (a) a greater improvement in second-trial sprint performance compared with placebo, (b) lower RPE immediately before the second sprint, and (c) higher post-exercise blood lactate concentrations following the second sprint. Taken together, these findings suggest that acute CM supplementation may modestly support repeated sprint performance under extended recovery conditions; however, the results should be interpreted with caution.

Both conditions showed faster sprint times in the second trial, suggesting that trial-related factors may have contributed to the observed performance changes. One possible explanation is that the first 100 m sprint and the repeated warm-up before the second trial together provided an additional preparatory stimulus. Previous studies have shown that warm-up can improve sprint performance through increases in muscle temperature, neuromuscular readiness, and metabolic activation [21,22]. Although post-activation performance enhancement (PAPE) has also been proposed as a possible contributor to short-term sprint performance enhancement [23,24,25], the relatively long recovery interval used in the present study makes its contribution uncertain. Therefore, the improvement observed from the first to the second sprint cannot be attributed solely to CM supplementation and may also reflect residual warm-up effects, familiarization, or trial-order influences. Nevertheless, the greater improvement observed in the CM condition compared with the placebo condition may suggest an additional supplementation-related effect beyond these general trial-related influences.

CM may promote NO production through conversion to arginine and may therefore influence physiological responses during repeated maximal sprinting [3]. However, because NO-related variables, ammonia, and muscle oxygenation were not measured in the present study, these potential mechanisms remain speculative and should not be interpreted as direct explanations for the present findings. At most, it is possible that CM ingestion may have contributed to a more favorable physiological state before the second sprint, but this cannot be confirmed by the current data. Accordingly, the present findings should be interpreted primarily in terms of the observed performance, perceptual, and lactate responses rather than specific underlying mechanisms.

In the present study, the CM condition resulted in higher post-exercise blood lactate concentrations following the second 100 m sprint than the placebo condition, which may reflect a greater glycolytic contribution during the repeated sprint effort. However, this finding should be interpreted cautiously. Elevated lactate concentration does not necessarily indicate improved glycolytic efficiency and may instead reflect a greater anaerobic metabolic load, which is not inherently advantageous for sprint performance or recovery. As highlighted in the previous literature, elevated blood lactate can represent greater metabolic stress rather than enhanced metabolic efficiency [26,27]. Therefore, although the increased lactate response in the present study may suggest altered metabolic involvement during the second sprint, its physiological significance remains uncertain.

In the present study, a notable finding was that the CM condition exhibited significantly lower RPE before the second sprint compared with the placebo condition. Similar tendencies have been reported previously, with meta-analytic evidence suggesting that citrulline supplementation may reduce perceived exertion during exercise [10]. These observations may indicate that CM supplementation influenced perceptual responses to the repeated sprint protocol. Although one possible explanation is altered ammonia handling or other fatigue-related processes, no markers of ammonia metabolism or central fatigue were assessed in the present study; therefore, such mechanisms remain speculative. More conservatively, the lower pre-sprint RPE observed in the CM condition may reflect reduced perceived fatigue or greater subjective readiness before the second sprint. This may have allowed participants to approach the second sprint with a lower perceived exertional state.

More broadly, previous studies have shown that the ergogenic effects of nutritional supplementation on repeated high-intensity efforts are highly context-dependent. For example, creatine-based supplementation has been shown to improve repeated sprint performance or power output in some exercise models [11,12], although the magnitude and consistency of these effects vary according to the protocol and recovery structure. In this context, acute CM ingestion may modestly support the repeated 100 m sprint performance across two maximal efforts separated by an extended recovery interval; however, the magnitude of this effect was modest. Although the absolute improvement in the second 100 m sprint was relatively small (≈0.17 s), even small differences may be meaningful in competitive sprinting, particularly among higher-level athletes for whom outcomes are often separated by small margins [28]. Nevertheless, the practical significance of these findings should be interpreted cautiously given the small sample size and the variability inherent to outdoor sprint testing. Although the present protocol does not directly replicate team-sport repeated sprint sequences, these findings may offer only limited preliminary insight into situations involving repeated maximal efforts separated by relatively long recovery intervals.

This study has several limitations. First, the sample size was relatively small (*n* = 11), and the participants were limited to male collegiate sprinters, which restricts the robustness and generalizability of the findings. In addition, sprint performance is subject to substantial inter-individual variability, and the small sample size may increase the risk of both Type I and Type II errors. Second, the effect size used for the a priori power analysis was derived from a resistance exercise study [9] rather than sprint performance, because no previous research has reported effect sizes for repeated 100 m sprints following citrulline supplementation. Consequently, the estimated sample size may not have fully captured the variability specific to sprint performance. Third, although environmental conditions were monitored and wind-adjusted sprint times were calculated, variability in outdoor testing conditions (e.g., temperature, humidity, and wind) may still have influenced sprint performance and physiological responses. Fourth, the supplementation protocol involved a single acute ingestion of 8 g CM, without comparison with lower doses or chronic supplementation strategies. Fifth, the study focused exclusively on acute effects; thus, potential long-term adaptations or effects in athletes from other sport disciplines were not assessed. Future research should include larger and more diverse participant samples and investigate both dose–response relationships and the chronic effects of CM supplementation. In addition, the inclusion of physiological markers may help to clarify the mechanisms potentially underlying the observed responses.

## 5. Conclusions

This study examined the effects of CM supplementation on repeated 100 m sprint performance in male collegiate sprinters. Compared with the placebo condition, CM ingestion was associated with a greater improvement in second-trial sprint performance, lower RPE before the second sprint, and higher post-exercise blood lactate concentrations. These findings suggest that acute CM supplementation may modestly support repeated sprint performance under extended recovery conditions. However, the results should be interpreted with caution given the modest effect size, the small sample size, and the potential influence of trial-order and environmental factors. Furthermore, the present conclusions apply only to the acute effects of a single 8 g dose within a controlled experimental setting. The potential effects of chronic supplementation, lower doses, or different athletic populations remain unknown and warrant further investigation.

## Data Availability

The data presented in this study are available from the corresponding author upon reasonable request. The data are not publicly available because they contain information that could compromise the privacy of the research participants.

## References

[1] Spencer M.R., Gastin P.B. (2001). Energy system contribution during 200- to 1500-m running in highly trained athletes. Med. Sci. Sports Exerc..

[2] Allen D.G., Lamb G.D., Westerblad H. (2008). Skeletal muscle fatigue: Cellular mechanisms. Physiol. Rev..

[3] Schwedhelm E., Maas R., Freese R., Jung D., Lukacs Z., Jambrecina A., Spickler W., Schulze F., Böger R.H. (2008). Pharmacokinetic and pharmacodynamic properties of oral L-citrulline and L-arginine: Impact on nitric oxide metabolism. Br. J. Clin. Pharmacol..

[4] Bradley S.J., Kingwell B.A., McConell G.K. (1999). Nitric oxide synthase inhibition reduces leg glucose uptake but not blood flow during dynamic exercise in humans. Diabetes.

[5] Moinard C., Nicolis I., Neveux N., Darquy S., Bénazeth S., Cynober L. (2008). Dose-ranging effects of citrulline administration on plasma amino acids and hormonal patterns in healthy subjects: The Citrudose pharmacokinetic study. Br. J. Nutr..

[6] Morita M., Hayashi T., Ochiai M., Maeda M., Yamaguchi T., Ina K., Kuzuya M. (2014). Oral supplementation with a combination of L-citrulline and L-arginine rapidly increases plasma L-arginine concentration and enhances NO bioavailability. Biochem. Biophys. Res. Commun..

[7] Suzuki T., Morita M., Kobayashi Y., Kamimura A. (2016). Oral L-citrulline supplementation enhances cycling time trial performance in healthy trained men: Double-blind randomized placebo-controlled 2-way crossover study. J. Int. Soc. Sports Nutr..

[8] Bailey S.J., Blackwell J.R., Lord T., Vanhatalo A., Winyard P.G., Jones A.M. (2015). L-citrulline supplementation improves O_2_ uptake kinetics and high-intensity exercise performance in humans. J. Appl. Physiol..

[9] Wax B., Kavazis A.N., Weldon K., Speriak J. (2015). Effects of supplemental citrulline malate ingestion during repeated bouts of lower-body exercise in advanced weightlifters. J. Strength Cond. Res..

[10] Rhim H.C., Kim S.J., Park J., Jang K.M. (2020). Effect of citrulline on post-exercise rating of perceived exertion, muscle soreness, and blood lactate levels: A systematic review and meta-analysis. J. Sports Health Sci..

[11] Bogdanis G.C., Nevill M.E., Aphamis G., Stavrinou P.S., Jenkins D.G., Giannaki C.D., Lakomy H.K.A., Williams C. (2022). Effects of oral creatine supplementation on power output during repeated treadmill sprinting. Nutrients.

[12] Zajac A., Golas A., Chycki J., Halz M., Michalczyk M.M. (2020). The Effects of long-term magnesium creatine chelate supplementation on repeated sprint ability (RAST) in elite soccer players. Nutrients.

[13] Mardokhi M., Rahimi M.R., Saedmocheshi S., Vasquez-Muñoz M., Andrade D.C. (2025). L-arginine supplementation does not enhance anaerobic performance in trained female handball players. J. Hum. Kinet..

[14] Faria V.S., Egan B. (2024). Effects of 3 days of citrulline malate supplementation on short-duration repeated sprint running performance in male team sport athletes. Eur. J. Sport Sci..

[15] McGawley K., Bishop D. (2015). Oxygen uptake during repeated-sprint exercise. J. Sci. Med. Sport.

[16] McKay A.K.A., Stellingwerff T., Smith E.S., Martin D.T., Mujika I., Goosey-Tolfrey V.L., Sheppard J., Burke L.M. (2022). Defining training and performance caliber: A participant classification framework. Int. J. Sports Physiol. Perform..

[17] Pérez-Guisado J., Jakeman P.M. (2010). Citrulline malate enhances athletic anaerobic performance and relieves muscle soreness. J. Strength Cond. Res..

[18] Mureika J.R. (2001). A realistic quasi-physical model of the 100 m dash. Can. J. Phys..

[19] Borg G. (1982). Psychophysical bases of perceived exertion. Med. Sci. Sports Exerc..

[20] Cohen J. (1988). Statistical Power Analysis for the Behavioral Sciences.

[21] Bishop D. (2003). Warm up I: Potential mechanisms and the effects of passive warm up on exercise performance. Sports Med..

[22] McGowan C.J., Pyne D.B., Thompson K.G., Rattray B. (2015). Warm-up strategies for sport and exercise: Mechanisms and applications. Sports Med..

[23] Seitz L.B., Haff G.G. (2016). Factors modulating post-activation potentiation of jump, sprint, throw, and upper-body ballistic performances: A systematic review with meta-analysis. Sports Med..

[24] Till K.A., Cooke C. (2009). The effects of postactivation potentiation on sprint and jump performance of male academy soccer players. J. Strength Cond. Res..

[25] Zois J., Bishop D., Ball K., Aughey R.L. (2011). High-intensity warm-ups elicit superior performance to a current soccer warm-up routine. J. Sci. Med. Sport.

[26] Brooks G.A. (2018). The science and translation of Lactate Shuttle theory. Cell Metab..

[27] Gladden L.B. (2004). Lactate metabolism: A new paradigm for the third millennium. J. Physiol..

[28] Trexler E.T., Persky A.M., Ryan E.D., Schwartz T.A., Stoner L., Smith-Ryan A.E. (2019). Acute effects of citrulline supplementation on high-intensity strength and power performance: A systematic review and meta-analysis. Sports Med..

